# Post-Vaccinal Encephalitis with Early Relapse after BNT162b2 (COMIRNATY) COVID-19 Vaccine: A Case Report

**DOI:** 10.3390/vaccines10071065

**Published:** 2022-07-01

**Authors:** Miguel A. Vences, Diego Canales, Maria Fe Albujar, Ebelin Barja, Mary M. Araujo-Chumacero, Edu Cardenas, Arturo Alvarez, Diego Urrunaga-Pastor

**Affiliations:** 1 Departamento de Neurología, Hospital Nacional Edgardo Rebagliati Martins, EsSalud, Lima 15072, Peru; diego.canales@upch.pe (D.C.); 075257@colegiomedico.org.pe (M.F.A.); ebelin.barja@unmsm.edu.pe (E.B.); mary.araujo@upch.pe (M.M.A.-C.); edu.cardenas@unmsm.edu.pe (E.C.); wilson.alvarez@upch.pe (A.A.); 2 Unidad para la Generación y Síntesis de Evidencias en Salud, Universidad San Ignacio de Loyola (USIL), Lima 15012, Peru; 3 Instituto de Evaluación de Tecnologías en Salud e Investigación—IETSI, EsSalud, Lima 14072, Peru

**Keywords:** encephalitis, COVID-19, vaccination, drug-related side effects and adverse reactions, SARS-CoV-2

## Abstract

We describe the case of a 72-year-old man who received the first dose of the BNT162b2 (COMIRNATY) vaccine against COVID-19 on 18 May 2021, and the second dose on 9 September 2021. One day after receiving the first dose, he cursed with malaise, headache, fever, confusion, aggressiveness, and gait alterations. We performed serum and cerebrospinal fluid (CSF) tests (finding elevated proteins in CSF) with negative results for infectious, systemic, and neoplastic causes. We performed brain nuclear magnetic resonance imaging (MRI), finding circumscribed encephalitis at the anterior frontal and bilateral temporal lobes. We were unable to perform a panel of antineuronal antibodies. The patient was readmitted due to early clinical relapse four days after receiving his second dose. We found sequelae lesions at the frontal level but with new demyelinating lesions at the left temporal level in brain MRI. We indicated methylprednisolone, and he presented a favorable improvement. We report an encephalitis case of probable autoimmune etiology after vaccination with BNT162b2, which presented early clinical relapse after receiving the second dose and presented a favorable response to methylprednisolone.

## 1. Introduction

COVID-19 is an infectious disease caused by the SARS-CoV-2 virus. As of 6 April 2022, more than 494 million cases and 6.2 million deaths have been recorded worldwide (1). Since the identification of the virus causing the pandemic, various vaccines against COVID-19 have been developed. To date, 11.2 billion doses have been administered worldwide, and it represents the best intervention to combat this disease and reduce the risk of hospitalizations and mortality [1,2,3].

The vaccines used globally are safe and effective, however, some nonspecific neurological adverse effects such as dizziness, headache, myalgia, and paresthesia have been reported [4]. Likewise, certain pathologies have been described post-vaccination, such as transverse myelitis, Guillain-Barré syndrome, or Bell’s palsy [5]. On the other hand, less frequent but severe complications, such as autoimmune encephalitis (AE), myoclonus, and acute disseminated encephalomyelitis, have been reported [6,7].

In previous case reports, the appearance of these neurological adverse effects has been described after the application of vaccines whose mechanism of action involves the mRNA, viral vector, and inactive virus [8]. Regarding the pathogenesis of vaccine-associated complications of mRNA COVID-19 vaccines, it has been proposed that the protein S expression by human cells (after translation of the mRNA vaccine) could trigger an inflammatory reaction, similar to that induced by the virus itself, producing these neurological complications [9].

We present the case of a patient with a clinical picture of possibly immune-mediated acute encephalopathy, with early relapse, in temporal association with the administration of the BNT162b2 (COMIRNATY) vaccine.

## 2. Case Report

We describe the case of a 72-year-old self-sufficient man with a history of type 2 diabetes mellitus and high blood pressure controlled with oral pharmacological treatment (850 mg of metformin three times per day and 10 mg of enalapril daily for the last five years). The patient self-reported no history of previous SARS CoV-2 infection and obtained a negative antigen test on hospital admission.

The patient received the first dose of the BNT162b2 (COMIRNATY) vaccine on 18 May 2021, with no apparent immediate discomfort. The next day, he developed nausea, vomiting, general malaise, left-sided headache, and fever, for which he went to the emergency room of a medical center, where they indicated symptomatic treatment. The patient’s clinical picture progressed during subsequent days, associating instability in the gait and episodes of confusion, not being able to recognize his relatives. For this reason, they went to the emergency room of a national hospital in Peru on 21 June 2021, with a four-week illness time.

**Figure 1:**
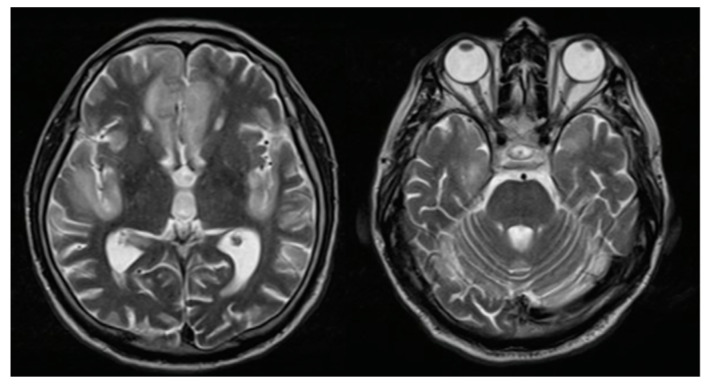
Brain magnetic resonance imaging: T2 sequence finding hyperintense lesions at the bilateral straight frontal gyri, left cingulate, and insula.

He was evaluated in the emergency room by the medical staff who requested computed tomography (CT) of the brain, evidencing a circumscribed hypodensity in the frontal lobe, deciding his hospitalization. At the clinical examination on admission, the patient was awake, confused, with a partial response to simple commands, without sensory or motor alterations and without signs of meningeal irritation. Due to acute encephalopathy of unidentified etiology, serum, imaging, and cerebrospinal fluid (CSF) tests were performed, ruling out a clinical picture of infectious, metabolic, collagen, or oncological origin. However, a high protein concentration (284 mg/dL) was highlighted, without cellularity or glucose consumption. We performed brain magnetic resonance imaging (MRI) with evidence of circumscribed encephalitis at the anterior frontal and bilateral temporal lobes (Figure 1). The patient received 10 mg/kg of daily intravenous acyclovir for seven days until we discarded a possible infectious cause.

During the hospital stay, the patient had a slow but favorable clinical evolution in the following two months, with results of brief cognitive tests in the lower range (Montreal Cognitive evaluation of 11 points). Due to the patient’s clinical evolution and without clear evidence of any etiology, he was discharged on 9 September 2021 with an outpatient follow-up indication.

The patient received the second dose of the BNT162b2 (COMIRNATY) vaccine against COVID-19 on the discharge day, presenting four days later a clinical picture similar to that of the previous episode, manifesting headache, nausea, vomiting, a heteroaggressive behavioral disorder, and episodes of environmental disconnection, for which he went again to the same hospital emergency room on 13 October 2021.

**Figure 2:**
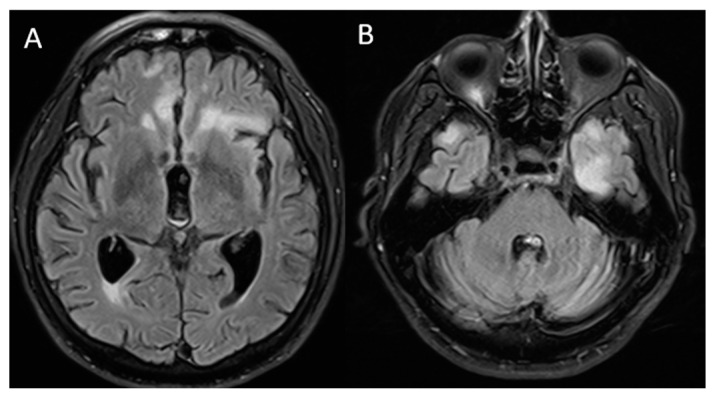
Brain magnetic resonance imaging: T2/FLAIR sequence finding (**A**) lower-volume lesions in the bilateral frontal lobes compared to that in the previous MRI and (**B**) new hyperintense lesions, predominantly in the left temporal region.

The patient was evaluated by a neurologist who requested new brain MRI, evidencing sequelae lesions at the frontal level, but with aggregation of new demyelinating lesions at the left temporal level (Figure 2). We performed serum tests and a lumbar puncture for suspicion of acute autoimmune encephalopathy (after ruling out a possible infectious process), finding proteinorrhachia (80 mg/dL). We started a five-day immunosuppressive treatment scheme with methylprednisolone at a dose of 1 g daily and immunoglobulin 0.4 g/kg daily.

The previously requested CSF and serum studies were negative for inflammatory, systemic autoimmune, neoplastic, and infectious disease. The cervical, thoracic, abdominal, and pelvic tomography with contrast, as well as the PET scan, excluded the presence of neoplasms.

In the two weeks after the treatment, the patient showed clinical improvement, without disconnection from the environment, but with residual neurocognitive disorder, characterized by failures in executive functions and with a Montreal Cognitive evaluation control result of 9 points.

After four weeks of treatment, we decided to start a new cycle of methylprednisolone similar to the previous regime and with subsequent treatment with prednisone at a dose of 1 mg/kg. We concluded the diagnosis of an early relapse of possibly immune-mediated post-vaccinal encephalitis, and he was discharged with subsequent outpatient follow-up with the indication of six-monthly cycles of methylprednisolone to avoid a possible new clinical relapse.

## 3. Discussion

AE in general and those associated with neuronal surface antibodies are rare diseases with an estimated annual incidence of 1 to 5 cases per million inhabitants [10]. AE post-vaccination against COVID-19 is a rare entity, and its incidence is not yet defined. In previous cases, the encephalitis symptoms’ appearance within three weeks after vaccination has been registered, defining this time as a relevant period [6,9,11,12,13,14,15] (Table 1). In our case, the patient presented a subacute onset of altered content of consciousness, confusion, did not recognize family members, presented episodes of aggressiveness and difficulty walking, which is comparable to that observed in previous cases of autoimmune encephalopathy [6,9,13].

Regarding the diagnosis, our patient underwent CSF and serum studies, which were negative for associated inflammatory, systemic autoimmune, neoplastic, and infectious disease. The cervical, thoracic, abdominal, and pelvic contrasted tomography and the PET scan excluded the presence of neoplasms. In the CSF, we found hyperproteinorrachia with negative cellularity, a result similar to that described in other cases of post-vaccination encephalitis [9,14]. However, in a case of post-vaccination NMDA-positive encephalitis with BNT162b2 (COMIRNATY), high cellularity with lymphocyte predominance was observed in the CSF [12].

Regarding brain MRI, in most of the previous cases, an image without alterations was presented [6,9,11,12,13], however, one case reported hyperintense images with edema at the hippocampal level [15]. On the other hand, in our case we found hyperintense lesions almost symmetrically. Concerning treatment, in most of the previous reports, methylprednisolone pulses were offered as the first treatment option, obtaining significant clinical improvement [6,9,11,12,14,15], as in our case.

The main limitation in our case report was not being able to perform an AE panel antibody dosage in our hospital, which has not allowed us to know if having received the COVID-19 vaccine was the primary trigger of encephalitis symptoms or was temporarily associated with the activity of an antineuronal antibody encephalitis. This limitation could be frequent in low-resource settings, producing an underreporting of AE in Latin America [16]. However, our case is consistent with previous reports, supporting our hypothesis, therefore, it could be important to report it despite not having an AE antibody panel. We support our probable AE diagnosis due to having criteria reported in a previous consensus [17,18]. In conclusion, we report the case of a patient with a clinical picture of encephalopathy of probable autoimmune etiology after vaccination with BNT162b2 (COMIRNATY) who presented an early clinical relapse after the second dose. More data is needed about the behavior of vaccination-associated encephalitis and management strategies to follow in these cases, even more so at this time, when a booster dose is an effective tool to reduce the risk of hospitalization for COVID-19.

## Figures and Tables

**Table 1:** Published cases reports of autoimmune encephalitis after vaccination against COVID-19.

Author (Year)	Age (Sex)	Comorbidities	Onset Clinical Picture	Antineuronal Antibodies	Cerebrospinal Fluid	Immunization History: Type (Laboratory) and Number of Doses	Time between Vaccination and Symptoms	Imaging Findings	Relapse	Type of Treatment	Maintenance Treatment
Vences M.A. (2022) In this study	72 (Male)	High blood pressure and diabetes mellitus	Headache, confusion, aggressiveness, instability	Not performed	Elevated protein levels	mRNA (Pfizer) and two doses	1st dose: 1 day and 2nd dose: 4 days	MRI: bilateral frontal and insular hyperintensity	Yes	Methylprednisolone, intravenous immunoglobulin	Six-monthly cycles of methylprednisolone
Abu-Riash A. (2021) [11]	20 (Male)	None	Fatigue, tonic-clonic seizures, hallucinations, bilateral hand tremor and amnesia	Not described	Normal	Inactivated virus (Sinopharm) and two doses	1 day	MRI: normal	No	Phenytoin, levetiracetam, teicoplanin, and methylprednisolone	Not described
Torrealba-Acosta G. (2021) [6]	77 (Male)	Coronary artery disease, hyperlipidemia, and hypothyroidism	Confusion, fever and generalized rash	Normal	Normal	mRNA (Moderna) and one dose	1 day	MRI: normal	No	Empiric broad-spectrum antibiotics, antiviral coverage, and methylprednisolone	Prednisone 60 mg daily for 3 weeks
Flannery P. (2021) [12]	20 (Female)	None	Motor dysfunction, a transient aphasia, decreased mentally acuity, insomnia, somatization of bowel and kidney disease	1:20 anti-NMDA	Mild lymphocyte pleocytosis with 12–14 nucleated cells/mm^3^	mRNA (Pfizer) and one dose	1 week	MRI and CT: normal	No	Intravenous immunoglobulin, methylprednisolone, rituximab	Not described
Takata J. (2021) [13]	22 (Female)	Non-syndromic retinitis pigmentosa	Confusion, visual, and tactile hallucinations	Negative	Pleocytosis	Viral vector (AstraZeneca) and two doses	3 weeks	MRI and CT: normal	No	Ceftriaxone, acyclovir, and olanzapine	Olanzapine 5 mg twice daily
H -T Fan (2022) [14]	22 (Male)	None	Fever, blurred vision, and seizures	Negative	Elevated protein level	mRNA (Moderna) and two doses	6 days	CT: normal, SPECT: mild hypoperfusion in the right temporal region	No	Levetiracetam, acyclovir, valproate sodium, and methylprednisolone	Not described
Hye-Rim Shin (2022) [15]	35 (Female)	Intellectual disability	Dysarthria, anxiety, and reduced voluntary movements	Negative	Normal	Viral vector (AstraZeneca) and one dose	5 days	MRI: mild swelling of the right hippocampus	No	Methylprednisolone, immunoglobulin, acyclovir, and rituximab	Not described
Al-Mashdali A. (2021) [9]	32 (Male)	None	Agitation, disorientation to time, place, person, and memory disturbances	Negative	Elevated protein levels	mRNA (Moderna) and one dose	2 days	MRI: normal	No	Ceftriaxone, acyclovir, and methylprednisolone	Not described

## Data Availability

The original contributions described in the case report are included in the article. Further inquiries can be directed to the corresponding author.
